# Pten inhibition dedifferentiates long-distance axon-regenerating intrinsically photosensitive retinal ganglion cells and upregulates mitochondria-associated Dynlt1a and Lars2

**DOI:** 10.1242/dev.201644

**Published:** 2023-04-24

**Authors:** Bruce A. Rheaume, Jian Xing, Agnieszka Lukomska, William C. Theune, Ashiti Damania, Greg Sjogren, Ephraim F. Trakhtenberg

**Affiliations:** ^1^ Department of Neuroscience, University of Connecticut School of Medicine, 263 Farmington Avenue, Farmington, CT 06030, USA; ^2^ The Jackson Laboratory for Genomic Medicine, Farmington, CT 06032, USA

**Keywords:** Axon growth, Optic nerve, Retinal ganglion cell, scRNA-seq, Mouse

## Abstract

Central nervous system projection neurons fail to spontaneously regenerate injured axons. Targeting developmentally regulated genes in order to reactivate embryonic intrinsic axon growth capacity or targeting pro-growth tumor suppressor genes such as *Pten* promotes long-distance axon regeneration in only a small subset of injured retinal ganglion cells (RGCs), despite many RGCs regenerating short-distance axons. A recent study identified αRGCs as the primary type that regenerates short-distance axons in response to Pten inhibition, but the rare types which regenerate long-distance axons, and cellular features that enable such response, remained unknown. Here, we used a new method for capturing specifically the rare long-distance axon-regenerating RGCs, and also compared their transcriptomes with embryonic RGCs, in order to answer these questions. We found the existence of adult non-α intrinsically photosensitive M1 RGC subtypes that retained features of embryonic cell state, and showed that these subtypes partially dedifferentiated towards an embryonic state and regenerated long-distance axons in response to Pten inhibition. We also identified Pten inhibition-upregulated mitochondria-associated genes, *Dynlt1a* and *Lars2*, which promote axon regeneration on their own, and thus present novel therapeutic targets.

## Teaser

**Summary:** A new method for capturing long-distance axon-regenerating RGCs for single-cell RNA-sequencing analysis reveals that adult neuronal subtypes that retain features of the embryonic cell state dedifferentiate in response to a treatment.

## INTRODUCTION

Mammalian central nervous system (CNS) projection neurons fail to spontaneously regenerate damaged axons (Curcio and Bradke, 2018; Williams et al., 2020). Several approaches succeeded in promoting various extents of axonal regeneration, for example, after optic nerve crush (ONC) (Benowitz et al., 2017; Chun and Cestari, 2017; Ghaffarieh and Levin, 2012; Williams et al., 2020; Kim et al., 2018; Park et al., 2008; Trakhtenberg et al., 2018; Moore et al., 2009). Nevertheless, even in the approaches targeting potent pro-growth tumorigenic factors, only a rare subset of the axons regenerates the full-length (Gokoffski et al., 2020; de Lima et al., 2012; Lim et al., 2016). A number of developmentally regulated genes have been found to underlie the developmental decline (Chen et al., 1995; Goldberg et al., 2002) in intrinsic capacity of retinal ganglion cells (RGCs) (and other CNS projection neurons; Poplawski et al., 2020) to grow axons (Apara et al., 2017; Blackmore et al., 2012; Trakhtenberg et al., 2014, 2018; Moore et al., 2009). However, the tumor suppressor gene, *Pten* is one of the most potent gene regulators of axon regeneration discovered to date. Pten suppresses axon regeneration through inhibition of the mTOR pathway (Park et al., 2008), and *Pten* knockout (KO) was shown to promote various extents of axon regeneration from the RGCs that included a subset of αRGCs (Duan et al., 2015; Jacobi et al., 2022). Although experimental gene therapy knockdown (KD) of *Pten* expression in adult RGCs promotes long-distance (i.e. at least the full length of the optic nerve) axon regeneration in a small subset of RGCs (Park et al., 2008; Kim et al., 2018; Yungher et al., 2015), it is concerning for clinical use (Keniry and Parsons, 2008), and safer downstream effectors of *Pten* KD and mTOR pathway regulation are being investigated (Duan et al., 2015; Li et al., 2016; Bray et al., 2019; Lukomska et al., 2021; Park et al., 2010). Here, we used a new method for capturing specifically the rare long-distance axon-regenerating RGCs for single-cell RNA-sequencing (scRNA-seq) analysis, and also compared their transcriptomes with embryonic RGCs. Our approach enabled us to: (1) investigate why, despite *Pten* KO in all RGC types, some do not regenerate axons, others (such as αRGCs) regenerate short-distance axons (Jacobi et al., 2022), and only a rare subset regenerates long-distance axons (Duan et al., 2015), and (2) characterize the relationship between long-distance axon regeneration promoted by targeting Pten and the developmental decline in intrinsic axon growth capacity.

## RESULTS

### Transcriptomic profiling of RGCs that regenerated long-distance axons in response to *Pten* KD

Multiple experimental approaches stimulate short-distance (i.e. up to 1.5 mm past the injury site) axon regeneration, but few stimulate long-distance axon regeneration (i.e. full-length of the optic nerve or longer). Moreover, even in the approaches which lead to long-distance axon regeneration, only a rare subset of RGCs regenerates axons 3 mm or longer, whereas the majority of the responding RGCs regenerate axons only a short-distance and then stall growth (Duan et al., 2015; Li et al., 2016; Bray et al., 2019; Kim et al., 2018). Because long-distance axon regeneration could provide insights into the mechanisms of full-length axon regeneration, we developed a novel surgical technique (that allows visual confirmation of appropriate targeting) for injecting CTB into the end of the optic nerve ∼3 mm distally from the injury site (as opposed to proximally, at 1.5 mm, as was done in another study; Jacobi et al., 2022), which enables retrograde labeling of the long-distance axon-regenerating RGCs, thereby prioritizing the identification of the rare subset of long-distance axon-regenerating RGCs over capturing many RGCs and mostly those that regenerate only short-distance axons.

**Fig. 1.**
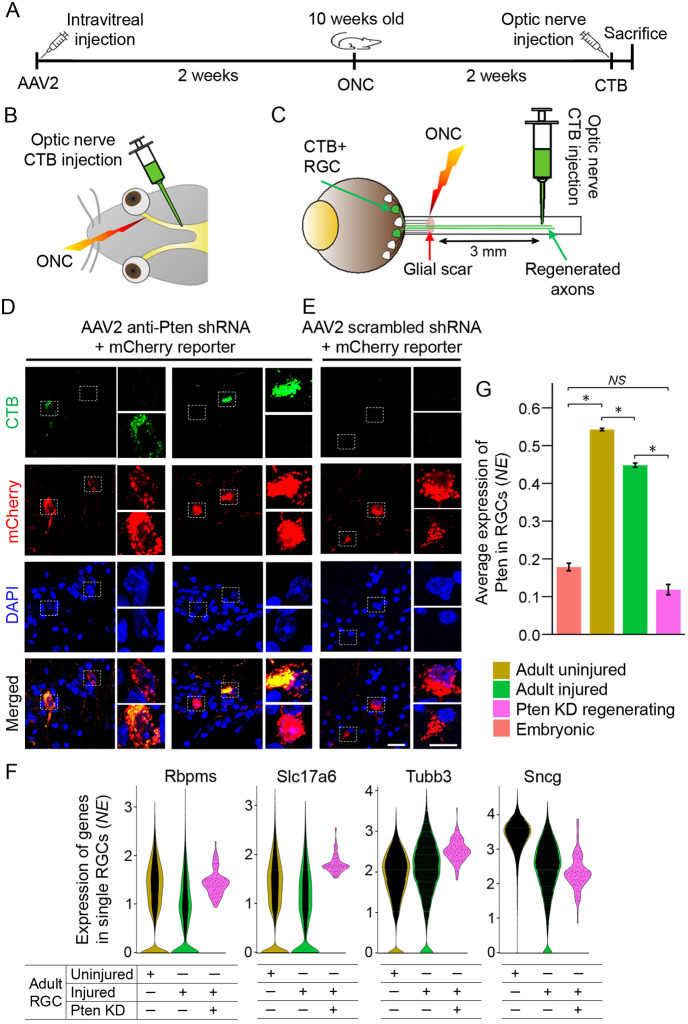
**Isolation of RGCs which regenerated long-distance axons in response to *Pten* KD.** (A-C) Experimental timeline (A; see Materials and Methods for details). Adult mice were pre-treated with intravitreally injected anti-Pten or scrambled shRNA (control) AAV2 viruses co-expressing mCherry reporter. Two weeks later, an ONC injury was performed. Two weeks after this, Alexa Fluor 488-conjugated CTB was injected into the end of optic nerve (3 mm distally from the ONC site) (B), and retrogradely transported via the regenerated axons into the RGC soma located in the retina (C). Twelve hours later, animals were sacrificed (A) either for histological analysis, or live RGCs were isolated by Thy1 immunopanning and FACS carried out for mCherry/CTB double-positive cells, which were immediately processed by droplet-based scRNA-seq. (D,E) Representative confocal images of the flat-mounted ganglion cell layer at 2 weeks after ONC, with CTB-488 injected into the end of the optic nerve 12 h before sacrifice, from the animals pre-treated with AAV2 anti-Pten shRNA (D) or AAV2 scrambled shRNA control (E). Only a small portion of anti-Pten shRNA AAV2-transduced RGCs expressing an mCherry reporter were also CTB^+^; examples of CTB^+^ and CTB^−^ RGCs expressing mCherry are shown (D). Examples of CTB^−^ (E) RGCs expressing mCherry reporter of transduction with scrambled shRNA control are also shown, but no CTB^+^ RGCs were found in the control condition. (F) Gene expression violin plots of pan-RGC marker genes in single cells, as marked, show that these markers are expressed in the *Pten* KD long-distance axon-regenerating RGCs in a similar range as in uninjured or injured (non-treated) adult RGCs. (G) *Pten* gene expression is substantially upregulated developmentally [from 0.18 normalized expression (NE) in embryonic to 0.54 NE in adult RGCs; *P*<0.001] but only modestly downregulated in RGCs after ONC (to 0.45 NE 2 weeks after injury; *P*<0.001). In the injured RGCs transduced with AAV2 anti-Pten shRNA (which regenerated long-distance axons after ONC), *Pten* gene expression is substantially knocked-down (to 0.12 NE; *P*<0.001) to below embryonic level (although not significantly different from embryonic; *P*=3.1). Data analyzed using one-way ANOVA, overall *F*=238.9, *P<0.001, with *P*-values of pairwise comparisons determined by posthoc LSD. Error bars represent s.e.m. NS, not significant. See Materials and Methods and Fig. 2 for more details on scRNA-seq analysis pipeline. Scale bars: 20 µm (D,E; main panels); 10 µm (D,E; insets).

Then, using scRNA-seq, we analyzed RGCs that responded to *Pten* KD by regenerating long-distance axons (i.e. full-length of the optic nerve, ∼3 mm from the injury site, or longer). *Pten* was knocked down using intravitreally injected adeno-associated virus serotype 2 (AAV2), which preferentially transduces RGCs and expresses anti-Pten short hairpin RNAs (shRNAs) known to promote axon regeneration after ONC (Yungher et al., 2015; Kim et al., 2018; Li et al., 2016; Ribeiro et al., 2020). Two weeks after ONC, long-distance axon-regenerating RGCs were isolated by fluorescence-activated cell sorting (FACS) from retinal cell suspension. At 12 h before sacrifice, Alexa Fluor-488-conjugated Cholera toxin subunit B (CTB) axonal tracer was injected into the optic nerve 3 mm distally from the ONC site. CTB was retrogradely transported to the RGC soma in the retina via long-distance regenerated axons. Only a small subset of the *Pten* KD-treated RGCs (identified by the expression of mCherry reporter) were CTB^+^ (Fig. 1A-D). No CTB^+^ RGCs were found in the retinas treated with the control vector expressing scrambled shRNAs and an mCherry reporter (Fig. 1E), as there were no regenerated axons in the control to uptake the CTB that was injected distally from the injury site. An mCherry reporter (identifying AAV2-transduced cells) and Alexa Fluor-488 (identifying long-distance regenerating RGCs) double-positive RGCs were isolated by FACS (Fig. 1A-E). The transcriptomes of 101 long-distance axon-regenerating RGCs (that passed quality control; see Materials and Methods) is a statistically appropriate representative sample of the target population comprised of only ∼90 RGCs per retina (representing 0.02% of the total retinal RGCs) that respond to *Pten* KD by regenerating long-distance axons at 2 weeks after ONC (Yungher et al., 2015; Kim et al., 2018) (101 is also comparable in number with 120 *Pten* KO short-distance axon-regenerating RGCs from a recent study; Jacobi et al., 2022).

**Fig. 2.**
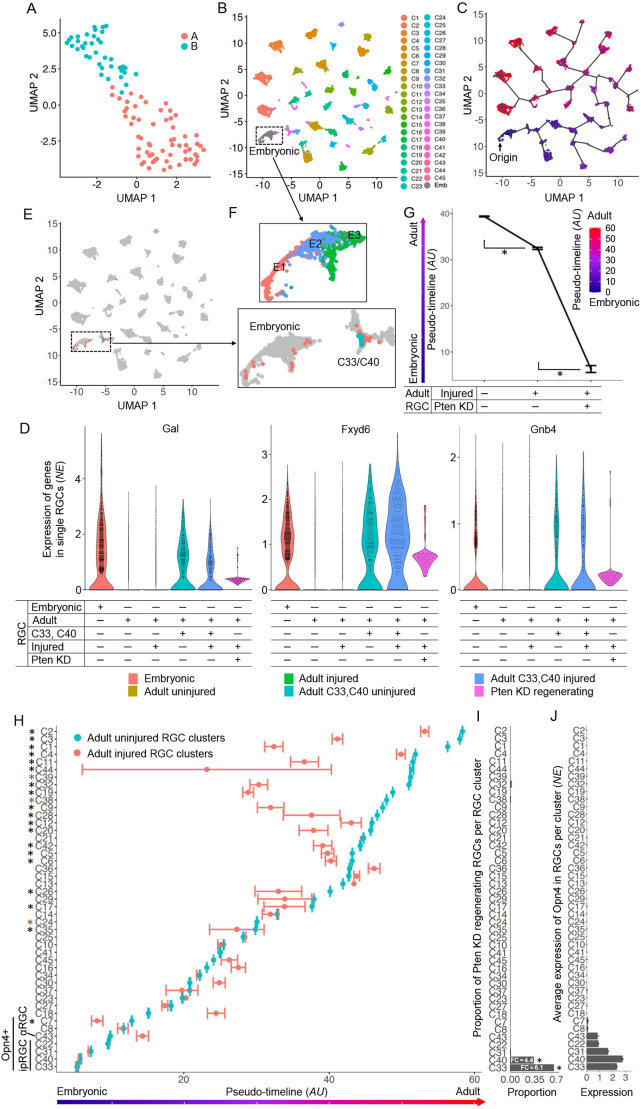
**Adult RGC subtypes that are more similar to embryonic RGCs regenerate long-distance axons and dedifferentiate towards an embryonic state.** (A) UMAP of *Pten* KD long-distance axon-regenerating RGCs shows segregation into two clusters (A and B, color-coded; see Materials and Methods for details). (B,C) UMAP of embryonic (gray; sub-clusters shown in inset F, upper panel) and adult RGC clusters (color-coded by cluster assignment per original publications; Tran et al., 2019; Rheaume and Trakhtenberg, 2022 preprint) (B), with a color-coded pseudo-timeline (C) indicating the relative transcriptomic changes progressing from embryonic into adult RGC state, and showing that within the adult RGC subtypes some remain more similar to embryonic whereas others change more during developmental maturation (i.e. localized further away from embryonic along the pseudo-timeline). Pseudo-timeline color-coded scale bar, in arbitrary units (AU), of relative distance along the pseudo-timeline is shown below panel C. (D) Gene expression violin plots of embryonic RGC marker genes in single cells, as marked, show that expression of these markers was retained in embryonic-like adult RGC subtypes C33/C40, as well as in the *Pten* KD long-distance axon-regenerating RGCs, but was downregulated in other RGC subtypes during maturation. (E,F) *Pten* KD long-distance axon-regenerating RGCs assignment to the UMAP of embryonic and adult atlas RGCs (from B,C; implementation of integration algorithm is detailed in the Materials and Methods) shows that almost all *Pten* KD long-distance axon-regenerating RGCs are similar to embryonic RGCs (i.e. bioinformatically mapped to embryonic RGCs and to clusters C33/C40, which are the closest to embryonic) (E). Magnified inset below (F, lower panel) shows that, long-distance regenerating RGCs cluster A (shown in A) mapped to the cluster C40 and embryonic RGCs, whereas long-distance regenerating RGCs cluster B (shown in A) mapped to cluster C33 (F). Assignments of *Pten* KD long-distance axon-regenerating RGCs by the integration algorithm only to the adult atlas RGC UMAP (without embryonic RGCs) results in almost all (96%) mapping only to the M1 ipRGC clusters C33/C40 (see H). (G) Averages of pseudo-timeline scores (from C and E) for individual RGCs comprising the adult atlas, injured and *Pten* KD long-distance axon-regenerating groups show that adult RGC transcriptomes partially revert towards an embryonic state after axonal injury, and that the long-distance regenerating RGC transcriptomes are significantly closer to the embryonic state than untreated injured RGC transcriptomes overall. Data are mean±s.e.m. Overall *F*=728.1, *P<0.0001 using one-way ANOVA, with pairwise comparisons by posthoc LSD. (H) Average pseudo-timeline scores (from C, per scale bar there) of the adult atlas RGC clusters (green), ranked lowest-to-highest on the embryonic-adult RGC pseudo-timeline (*x*-axis; the lower the score the closer to embryonic state the cluster is). Of the surviving RGC clusters (red), 19 shifted closer to the embryonic state by 2 weeks after injury (indicated by black asterisk, *P<*0.0001) based on their average pseudo-timeline scores (shown on the *x*-axis), but still not as close as uninjured ipRGC clusters C33/C40 (which regenerated long-distance axons in response to *Pten* KD treatment, see I). All RGC atlas Opn4^+^ clusters, which include all known ipRGC amongst other clusters, are the closest to the embryonic state on the pseudo-timeline, as indicated on the *y*-axis (see J). Data are mean±s.e.m. shown for clusters; overall *F*=190.8, *P<*0.0001 using two-way ANOVA, with pairwise comparisons by posthoc LSD at *P*<0.05 showing cluster means which shifted significantly closer to the embryonic state (indicated by black asterisk). Clusters C24, C38 and C39 did not survive for more than 2 weeks after injury (brown asterisk). (I) Compared with cluster proportion in the adult RGC atlas, the proportion of *Pten* KD long-distance axon-regenerating RGCs (which bioinformatically mapped to adult atlas RGC clusters) is significantly enriched in the adult RGC clusters that are the closest to the embryonic RGC state (on the embryonic-adult RGC state pseudo-timeline, *x*-axis in H; *P<0.05 by EdgeR, see Materials and Methods). (J) Opn4 expression is enriched in RGC atlas clusters C7, C8, C43, C22, C31, C40 and C33 (of which the last four are ipRGC clusters) consistent with the original publication (Tran et al., 2019). These Opn4^+^ clusters are the closest to the embryonic state on the pseudo-timeline, as indicated in panel H. Error bars represent s.e.m. NE, normalized expression.

We compared the transcriptomes of RGCs that regenerated long-distance axons in response to *Pten* KD with adult uninjured and injured RGC scRNA-seq transcriptomes (Tran et al., 2019; Rheaume and Trakhtenberg, 2022 preprint). The long-distance axon-regenerating RGCs expressed canonical pan-RGC markers (*Rbpms*, *Slc17a6*, *Tubb3* and *Sncg*) in a similar range as uninjured or injured (non-treated) RGCs (Fig. 1F). We then analyzed developmental regulation and KD efficiency of *Pten* gene expression, by comparing adult *Pten* KD-treated and untreated with embryonic RGCs (Lo Giudice et al., 2019). Expression of *Pten* was substantially upregulated during RGC maturation from embryonic to adult, and KD of *Pten* by AAV2 expressing anti-Pten shRNAs substantially reduced *Pten* expression in the *Pten* KD long-distance axon-regenerating RGCs to below embryonic level (Fig. 1G). The *Pten* KD long-distance axon-regenerating RGCs also segregated into two clusters (Fig. 2A).

### RGC subtypes retaining features of an embryonic state are present in the adult retina

The embryonic retina contains RGCs at various stages of development, as RGCs are born on different days in the embryonic retina (Prasov and Glaser, 2012; Bhansali et al., 2014; Lo Giudice et al., 2019). Together with the adult RGC scRNA-seq atlas (Tran et al., 2019; Rheaume and Trakhtenberg, 2022 preprint), the embryonic RGC scRNA-seq dataset (Lo Giudice et al., 2019) enabled generation of a developmental pseudo-timeline spanning the progression from embryonic into adult cell states (Fig. 2B,C). In analyzing the relative transcriptomic changes progressing from embryonic into adult RGC state (along the developmental pseudo-timeline), we unexpectedly found that some adult RGC subtypes remain more similar to the embryonic state, whereas others change more during maturation (Fig. 2C). We then identified marker genes, *Gal*, *Fxyd6* and *Gnb4*, that are expressed in both embryonic RGCs and in adult RGC subtypes C33/C40 that are the most similar to embryonic RGCs, which further supports the existence of adult subtypes that retained features of embryonic cell state (Fig. 2D).

### Long-distance axon regeneration promoted by *Pten* KD is associated with dedifferentiating RGCs towards an embryonic state

We then bioinformatically mapped (see Materials and Methods) the *Pten* KD long-distance regenerating RGCs to the UMAP of embryonic and adult RGC atlas, and found that almost all *Pten* KD long-distance regenerating RGCs were assigned to embryonic RGCs and to adult M1 intrinsically photosensitive (ip) RGC clusters C33/C40 that are the closest to embryonic RGCs (Fig. 2E). Specifically, *Pten* KD long-distance regenerating RGC cluster A mapped to cluster C40 and embryonic RGCs, whereas *Pten* KD long-distance regenerating RGC cluster B mapped to cluster C33 (Fig. 2F). When the *Pten* KD long-distance axon-regenerating RGCs are bioinformatically mapped only to the adult atlas RGC UMAP (without embryonic RGCs), almost all (96%) are assigned only to the non-α M1 ipRGC clusters C33/C40. By contrast, using the same method to bioinformatically map the *Pten* KO mostly short-distance axon-regenerating RGCs to the atlas RGC UMAP, the cells are assigned primarily to non-ip αRGCs, consistent with the original report from which the scRNA-seq dataset on the *Pten* KO short-distance axon-regenerating RGCs was obtained (Jacobi et al., 2022) (Fig. S1). Modest upregulation of only one αRGC marker Spp1 in the C33/C40 *Pten* KD long-distance axon-regenerating is consistent with *Pten* KO upregulating Spp1 (Kim et al., 2014; Sato et al., 2006; Lu et al., 2013) (Fig. S1D).

We also found that transcriptomes of untreated adult RGCs overall, and particularly transcriptomes of 19 clusters that survived 2 weeks after injury (Rheaume and Trakhtenberg, 2022 preprint) (Fig. 2G,H), partially reverted towards embryonic state, but not as close as the *Pten* KD long-distance regenerating RGC transcriptomes (which are the closest to the embryonic state; Fig. 2G). Moreover, C33/C40 subtypes to which the *Pten* KD long-distance axon-regenerating RGCs mapped, are the closest to embryonic state even in an uninjured state (Fig. 2H), and the proportion of *Pten* KD long-distance regenerating RGCs that mapped to these clusters is substantially enriched compared with the proportion of C33/C40 in the RGC atlas (Fig. 2I).

### RGCs that regenerate long-distance axons in response to *Pten* KD originate from the embryonic-like adult RGC subtypes

The existence of subtypes that retained features of embryonic cell state raises the possibility that they might be the RGCs which regenerated long-distance axons in response to *Pten* KD, particularly considering that the long-distance regenerating RGCs transcriptomically mapped almost exclusively to these embryonic-like adult RGC subtypes. C33/C40 are M1 ipRGCs within a subset of Opn4^+^ clusters – C7, C8, C43, C22, C31, C40 and C33 – the last four of which are ipRGCs (Tran et al., 2019). Although all Opn4^+^ clusters (Fig. 2J) are closer to embryonic state on the pseudo-timeline compared with all other clusters (Fig. 2H), the RGCs which regenerated long-distance axons in response to *Pten* KD mapped to the two closest to embryonic state ipRGCs C33/C40 (Fig. 2H,I). *Pten* KD long-distance axon-regenerating RGCs mapping to C33/C40 is partially consistent with the hypothesis that RGC types that are more resilient to injury (Pérez de Sevilla Müller et al., 2014; Tran et al., 2019; Rheaume and Trakhtenberg, 2022 preprint) are more responsive to Pten inhibition for regenerating axons (Li et al., 2016; Duan et al., 2015; Bray et al., 2019), but a recent study (Jacobi et al., 2022) identified αRGCs as the primary type responding to *Pten* KO by regenerating at least short-distance axons (Fig. S1). However, RGCs which regenerated long-distance axons in response to *Pten* KD appear to have originated from the non-α ipRGC subtypes C33/C40. It is also possible that other RGC subtypes responded to *Pten* KD but dedifferentiated towards an embryonic state (which facilitated long-distance axon regeneration) and thus transcriptomically mapped to embryonic-like C33/C40.

**Fig. 3.**
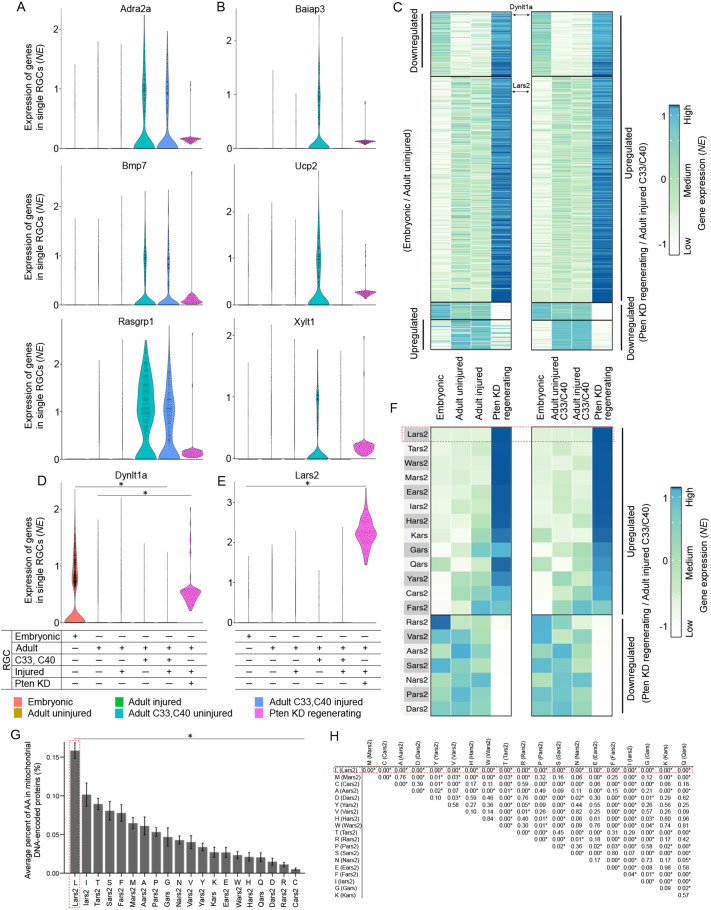
**Developmentally regulated and non-regulated DEGs in the *Pten* KD long-distance axon-regenerating RGCs.** (A,B) Gene expression violin plots of clusters C33/C40 marker genes show that markers were upregulated during RGC maturation only in these, but not in other, adult RGC subtypes, and that these markers are expressed in the *Pten* KD long-distance axon-regenerating RGCs (A). Some of these developmentally upregulated C33/C40 marker genes were downregulated by injury in (non-treated) adult RGCs, as marked, but injury-induced downregulation of their expression was rescued in the *Pten* KD long-distance axon-regenerating RGCs (B). (C) Heatmap of genes which are differentially expressed between clusters C33/C40 of ONC RGCs and *Pten* KD long-distance axon-regenerating RGCs (upregulated: log2 fold change (FC) ≥1.5 and *Pten* KD RGC expression ≥0.5 NE; downregulated: log2 FC ≤−1.5 and ONC RGC expression ≥0.5 NE). The genes displayed in the top-most and bottom-most sections of the heatmap also demonstrate a developmentally regulated pattern. Developmentally upregulated genes are those which also have a log2 FC ≥0.5 between embryonic and adult clusters C33 and C40 and ≥0.01 NE in the adult C33 and C40 clusters. Developmentally downregulated genes: log2 FC ≤−1.5 and ≥0.01 NE in the embryonic timepoint. All gene FCs displayed in the heatmap are significantly differentially expressed (*P<*0.05; Mann–Whitney *U-*test). (D) *Dynlt1a* gene expression is substantially downregulated developmentally (from 0.4 NE in embryonic to 0.02 NE in adult RGCs; *P*<0.001) and does not change after ONC. However, in the *Pten* KD long-distance axon-regenerating RGCs, *Dynlt1a* gene expression is substantially upregulated (to 0.55 NE; *P*<0.001) to even above embryonic level. Data analyzed using one-way ANOVA, overall *F*=1756.3, *P*<0.0001, with *P*-values of pairwise comparisons determined by posthoc LSD. Significant differences (*P<*0.001) indicated by an asterisk. (E) *Lars2* gene is expressed at a relatively low basal level and is not regulated developmentally or after ONC (ranging from 0.04-0.05 NE through development and after injury). However, in the *Pten* KD long-distance axon-regenerating RGCs, *Lars2* gene expression is substantially upregulated (to 2.24 NE; *P*<0.001). Data analyzed using one-way ANOVA, overall *F*=2334.9, *P*<0.0001, with *P*-values of pairwise comparisons determined by posthoc LSD. Significant differences (*P<*0.001) indicated by an asterisk. (F) Heatmap of mt-tRNA-specific aaRSs genes, which are differentially expressed between clusters C33/C40 of ONC RGCs and Pten KD long-distance axon-regenerating RGCs (downregulated and upregulated, with *Lars2* being the most highly upregulated, as marked). (G,H) Average percent of amino acids (AAs) in all mtDNA-encoded proteins, ranked from the highest (left) to lowest (right), showing that Lars2-aminoacylated L (outlined with dashed line) is significantly more enriched than any other AA in all mtDNA-encoded proteins. Error bars represent s.e.m. (G). Data analyzed using ANOVA, overall *F*=21.4, *P<0.001, with *P*-values for pairwise comparisons determined by posthoc LSD, showing that L is the only AA that is significantly (*P*<0.001) overrepresented in mitochondrial DNA-encoded proteins compared with every other AA (and not just compared with some AAs) (H). NE, normalized expression.

To resolve between these possibilities, we analyzed whether *Pten* KD long-distance axon-regenerating RGCs are enriched only for C33/C40 markers that are also enriched in embryonic RGCs (which could be a consequence of any subtype upregulating embryonic genes during dedifferentiation), or whether they are also enriched for C33/C40 markers that are unique to a mature cell state and not expressed in embryonic RGCs (which would suggest origination from C33/C40). We found that a substantial portion of the differentially expressed genes (DEGs) in the *Pten* KD long-distance regenerating RGCs partially reverted their levels of expression towards an embryonic RGC state (Table S1, and see Fig. 3C), while retaining the expression of embryonic RGC and embryonic-like adult RGC subtype markers, *Gal*, *Fxyd6* and *Gnb4* (Fig. 2D). We also found that C33/C40 markers not expressed in embryonic RGCs (Rheaume and Trakhtenberg, 2022 preprint) are enriched in the *Pten* KD long-distance axon-regenerating RGCs, namely *Adra2a*, *Bmp7* and *Rasgrp1*, as well as *Baiap3*, *Ucp2* and *Xylt1*, injury-induced downregulation of which was rescued by *Pten* KD (Fig. 3A,B). These data suggest that the long-distance axon-regenerating RGCs are more similar to embryonic-like C33/C40 because they originated from these subtypes, and that this is not a consequence of dedifferentiation from other subtypes.

### Developmentally regulated and non-regulated genes are differentially expressed in long-distance axon-regenerating RGCs

Next, we identified groups of up- and downregulated genes, which partially reverted their expression towards an embryonic RGC state, as well as those that are not developmentally regulated (Fig. 3C and Table S1). Axon regeneration may require recapitulation of the molecular mechanisms involved in developmental embryonic axon growth (Filbin, 2006; Hilton and Bradke, 2017; Yaniv et al., 2012; Harel and Strittmatter, 2006; Goldberg et al., 2002; Chen et al., 1995; Poplawski et al., 2020; Moore et al., 2009). However, the adult CNS environment is different from embryonic and is further altered by lesion. For example, the glial scar and immune cells (that responded to lesion) were not present along the developmental axonal path, and therefore axon regeneration may also require targeting the molecular mechanisms that were not involved in developmental axon growth (Kim et al., 2018; Chen et al., 2000; Shen et al., 2009; Geoffroy and Zheng, 2014; Low et al., 2008; Yiu and He, 2006). DEG analysis of *Pten* KD long-distance axon-regenerating RGCs revealed patterns of gene expression that are consistent with both approaches (Fig. 3C). Therefore, we selected representative DEG candidates consistent with each approach for testing in the axon regeneration assay.

### Mitochondria-associated *Dynlt1a* and *Lars2* are enriched in long-distance axon-regenerating RGCs

We selected *Dynlt1a*, which reverted its expression towards an embryonic RGC state (Fig. 3D), and the non-developmentally-regulated *Lars2*, which was upregulated in long-distance axon-regenerating RGCs but otherwise expressed at a relatively low basal level across conditions (Fig. 3E). We focused on these genes, because they could regulate mitochondrial dynamics involved in axonal regeneration (Luo et al., 2016; Han et al., 2016; Kreymerman et al., 2019; Zhou et al., 2016; Cartoni et al., 2016; Cheng et al., 2022). Dynlt1a is involved in bi-directional cargo (e.g. mitochondria; Fang et al., 2011) transport (Ligon et al., 2004) and regulates neurite growth in culture (Chuang et al., 2005). Lars2 is a nuclear DNA (nucDNA)-encoded aminoacyl-tRNA synthetase (aaRS), required for translation of mitochondrial DNA (mtDNA)-encoded proteins by aminoacylation with Leucine (L) to make the mtDNA-encoded tRNA-L (Sohm et al., 2004). MtDNA encodes the full set of tRNAs required for translation of mtDNA-encoded genes (which are not compensated for by the nucDNA-encoded tRNAs), and nucDNA encodes the full set of aaRSs that aminoacylate mtDNA-encoded tRNAs with their cognate amino acids (which are not compensated for by the aaRSs that aminoacylate the nucDNA-encoded tRNAs) (González-Serrano et al., 2019; Wang et al., 2021). We found that all aaRSs specific to the mt-tRNAs are differentially expressed in the *Pten* KD long-distance regenerating RGCs, with *Lars2* being the most highly upregulated (Fig. 3F). Moreover, Lars2-aminoacylated L is significantly more enriched than any other amino acid in all 13 mtDNA-encoded proteins (Fig. 3G,H). Therefore, we hypothesized that involvement of Lars2 and Dynlt1a in regulation of axonal mitochondrial dynamics may render them plausible downstream effectors of *Pten* KD for promoting axon regeneration.

### Dynlt1a and Lars2 promote axon regeneration after optic nerve injury

**Fig. 4.**
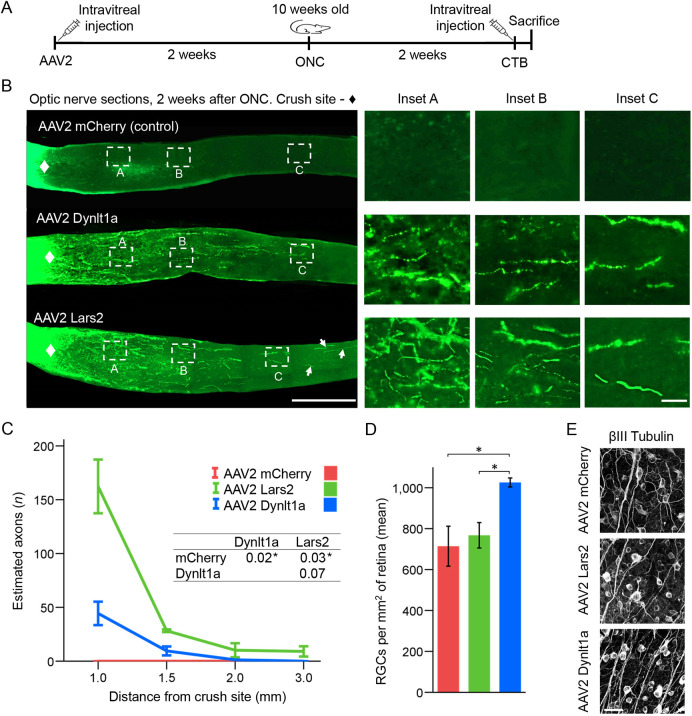
**Dynlt1a and Lars2 promote axon regeneration and RGC survival.** (A) Experimental timeline: 8-week-old mice were pre-treated with AAV2 vectors expressing *Dynlt1a*, *Lars2* or mCherry control. ONC injury was performed 2 weeks later. Animals were sacrificed for histological analysis 2 weeks after ONC. Axonal tracer CTB was injected intravitreally before sacrifice. (B) Representative images of the optic nerve longitudinal sections with CTB-labeled axons at 2 weeks after ONC from the animals pre-treated with AAV2 expressing *Dynlt1a*, *Lars2* or mCherry control, as marked. The edges of the tissue were optically trimmed (i.e. cropped-out) due to artefactual autofluorescence that is common at tissue edges. Insets show representative images of the optic nerve regions proximal and distal to the injury site, magnified for better visualization of the axons or their absence. The longest regenerating (by 2 weeks after ONC) axons in the *Lars2*-treated condition are indicated by arrows. (C) Quantitation of CTB-labeled regenerating axons at 2 weeks after ONC, at increasing distances from the injury site, after pre-treatment with AAV2 expressing *Dynlt1a*, *Lars2* or mCherry control, as marked (mean±s.e.m.; *n*=4 cases per group). Data analyzed using repeated measures ANOVA, sphericity assumed, overall *F*=13.4, *P*<0.001, with *P*-values of pairwise comparisons determined by posthoc LSD shown in the inset table, and significant differences (*P*<0.03) indicated by an asterisk. (D,E) Quantitation of RGC survival in retinal flatmounts immunostained for an RGC marker βIII-Tubulin (Tuj1 antibody) at 2 weeks after ONC, pre-treated with AAV2 expressing *Dynlt1a*, *Lars2* or mCherry control (mean±s.e.m.; *n*=4 cases per group). Data analyzed using ANOVA, overall *F*=5.6, *P*<0.04, with *P*-values of pairwise comparisons determined by posthoc LSD. Significant differences (*P*<0.03) indicated by an asterisk (D). Corresponding representative images are shown (E). Scale bars: 500 µm (B, main panels); 50 µm (B, insets; E).

We then tested whether Dynlt1a and Lars2 are sufficient to promote axon regeneration, using an established assay (Park et al., 2008; Kim et al., 2018; Yungher et al., 2015; Lukomska et al., 2021; Trakhtenberg et al., 2018). AAV2 vectors expressing *Dynlt1a*, *Lars2* or mCherry control, were injected intravitreally. ONC was performed 2 weeks later. To visualize the regenerating axons or their absence, CTB was injected 1 day before sacrifice at 2 weeks after ONC. The number of regenerating axons and RGC survival was quantified (see Materials and Methods for details; experimental timeline in Fig. 4A). We found that Dynlt1a and Lars2 promoted axon regeneration at least 2 mm and 3 mm past the injury site, respectively, compared with only minor axonal sprouting (as expected) in control (Fig. 4B,C). No spared axons were detected in either group. Dynlt1a (but not Lars2) also promoted RGC survival compared with the control group (Fig. 4D,E).

### A gene network involving *Lars2* and *Dynlt1a* shows the association of mitochondrial and axonal growth biological processes

**Fig. 5.**
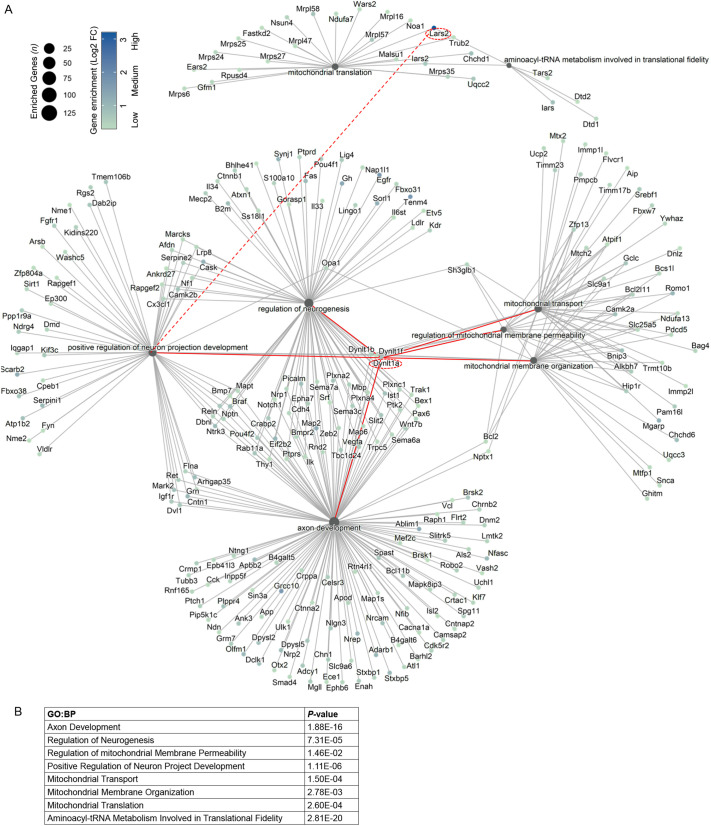
**Convergence of mitochondrial and axonal growth biological processes in the gene network upregulated in *Pten* KD long-distance axon-regenerating RGCs.** (A) Gene-Concept Network Plot of a subset of genes co-upregulated in response to *Pten* KD in long-distance axon-regenerating RGCs, shows association between the gene-ontology biological processes (GO:BP) of mitochondria, axonal growth and neurodevelopment, which involve *Lars2* and *Dynlt1a* (red lines). The finding that *Lars2*, which belongs to the GO:BP ‘mitochondrial translation’, promotes axon regeneration, which belongs to the GO:BP ‘positive regulation of neuronal axonal projection’, has linked (red dashed line) these biological processes. Bubble size scale indicate GO:BP node size based on the number of upregulated genes within that node. Color scale bar indicates the fold change (FC) of each gene in Pten KD long-distance axon-regenerating RGCs relative to injured untreated RGCs (see Materials and Methods for more details). (B) Functional enrichment analysis *P*-values of the GO:BP terms shown in Gene-Concept Network Plot. Terms are ordered by the decreasing number of DEGs in *Pten* KD long-distance axon-regenerating RGCs for respective GO:BP term.

To gain further insight into the mechanisms through which Lars2 and Dynlt1a promote axon growth, we analyzed the gene network upregulated in the *Pten* KD long-distance axon-regenerating RGCs. We found that the biological processes most co-enriched in the gene network (involving *Lars2* and *Dynlt1a*) upregulated in the *Pten* KD long-distance axon-regenerating RGCs were related to mitochondria, axonal growth and neurodevelopment (Fig. 5A,B). Furthermore, our finding that Lars2 promotes axon regeneration, which belongs to the gene ontology biological process (GO:BP) ‘positive regulation of neuronal axonal projection’, has linked it to the GO:BP ‘mitochondrial translation’ (under which *Lars2* was previously annotated) (Fig. 5A,B). Thus, a cross-talk between mitochondrial translation and axonal growth processes, and involvement of a co-upregulated gene network, may underlie our finding that Lars2 and Dynlt1a promote axon regeneration independently from each other.

## DISCUSSION

The failure of CNS projection neurons to spontaneously regenerate long-distance axons after injury or in neurodegenerative disease presents a major medical problem (Curcio and Bradke, 2018; Williams et al., 2020), as even in the approaches targeting clinically-concerning tumorigenic factors, only a rare subset of axons regenerate the full-length in animal models (Gokoffski et al., 2020; de Lima et al., 2012; Lim et al., 2016). To tackle this problem, studies have shown that the intrinsic axon growth capacity, which declines during maturation in the mammalian CNS (Goldberg et al., 2002), can be reactivated to some extent by targeting neuronal developmentally regulated genes (e.g. *Klf4*, *Klf9*) (Trakhtenberg et al., 2018; Moore et al., 2009), whereas axonal injury itself tilts the transcriptome of adult CNS neurons towards an embryonic state (Poplawski et al., 2020) (although failing to elicit axon regeneration). On the other hand, targeting the tumor suppressor Pten (Park et al., 2008) promotes various extents of axon regeneration, mostly short-distance axons from αRGCs and other RGC types (Jacobi et al., 2022), but also long-distance (i.e. full-length of the optic nerve or longer) regeneration from a rare subset of α and/or ip RGC subtypes (Duan et al., 2015).

Here, we demonstrate that, although bulk-RNA-seq has previously left unresolved whether injury itself tilts the transcriptome of all or only some adult CNS neuronal subtypes towards an embryonic state (Poplawski et al., 2020), scRNA-seq-enabled cluster-specific analysis revealed that only a subset of RGC subtypes reverts their transcriptomes towards an embryonic state. However, marginal dedifferentiation by injury alone fails to elicit spontaneous axon regeneration (as although neurons attempt, they fail to regenerate axons after injury; Sellés-Navarro et al., 2001). Unexpectedly, we also found the existence of adult RGC subtypes that retained features of an embryonic cell state, and identified *Gal*, *Fxyd6* and *Gnb4* as marker genes that are expressed in embryonic RGCs and then downregulated during maturation in all RGC subtypes, except a subset of Opn4^+^ RGCs, primarily subtypes C33 and C40 (that are transcriptomically more similar to embryonic RGCs than any other RGC subtype). We then showed that Pten inhibition-promoted long-distance (i.e. full-length of the optic nerve or longer) axon regeneration is associated with partial dedifferentiation towards an embryonic state of the responding M1 ipRGCs from subtypes C33 and C40. It is possible that these subtypes are able to respond to *Pten* KD by regenerating axons long-distance, because during maturation they retained features of an embryonic cell state (in contrast to other RGC subtypes), which enhanced their axon regeneration response to *Pten* KD. It would be important to investigate in future studies: (1) whether features of an embryonic cell state retained in the C33 and C40 subtypes are necessary for their enhanced axon regeneration response to *Pten* KD; (2) whether these features in the C33 and C40 subtypes also enhance axon regeneration promoted by other treatments (e.g. *Klf9* KD); (3) whether experimentally reinstating features of an embryonic cell state in other RGC subtypes would also enhance their axon regeneration in response to *Pten* KD or other treatments.

We also identified novel mitochondrial factors involved in axon regeneration (Luo et al., 2016; Han et al., 2016; Kreymerman et al., 2019; Zhou et al., 2016; Cartoni et al., 2016; Cheng et al., 2022). Dynlt1a is involved in bi-directional axonal transport of mitochondria along microtubules (Fang et al., 2011; Ligon et al., 2004; Chuang et al., 2005), which may be needed to supply mitochondria-generated energy (and ‘building materials’) for assembling the regenerating axonal segments. We found that Lars2-aminoacylated L (to mt-tRNA-L) is the most prevalent amino acid in (and therefore Lars2 is a limiting factor for production of) all mtDNA-encoded proteins, and Lars2 is the most highly upregulated mt-tRNA-specific aaRS in the Pten regenerating RGCs. Thus, Lars2 may enable synthesis of axonal mitochondria that are needed to supply energy for assembling the regenerating axonal segments. We also provided further insight into the mechanisms through which Lars2 and Dynlt1a promote axon growth, by showing that the biological processes most co-enriched in the gene network (which involved *Lars2* and *Dynlt1a*) upregulated in the *Pten* KD long-distance axon-regenerating RGCs were related to mitochondria, axonal growth and neurodevelopment. Furthermore, our finding that Lars2 promotes axon regeneration, which belongs to the gene-ontology biological processes (GO:BP) ‘positive regulation of neuronal axonal projection’, has linked this biological process to the GO:BP ‘mitochondrial translation’ (under which *Lars2* was previously annotated). Thus, a cross-talk between mitochondrial translation and axonal growth processes, and involvement of a subset of co-upregulated gene network, may underlie the ability of Lars2 or Dynlt1a to promote axon regeneration independently from each other. It will be important to investigate the roles of Lars2 and Dynlt1a in mitochondrial dynamics of regenerating axons, and whether co-targeting Lars2 and Dynlt1a will lead to a more robust axon regeneration than targeting each factor alone. Future studies also need to test whether over the long-term (i.e. longer than 2 weeks after ONC) axon regeneration achieved by Lars2 and Dynlt1a has the potential to regenerate beyond the optic chiasm, through the optic tract, and potentially to the postsynaptic target neurons in respective brain regions.

Our experimental approach prioritized identification of the rare subset of RGCs that regenerates long-distance (∼3 mm from the ONC site or longer) axons, rather than capturing many RGCs, and mostly those that regenerate axons only over a short distance (up to 1.5 mm from the ONC site). In order to reliably inject axonal tracer into the end of the optic nerve 3 mm beyond the injury site, we developed a surgical technique for appropriate targeting with visual confirmation (see Materials and Methods). Our approach yielded insights into long-distance axon regeneration that are more relevant for providing clues to developing axon regeneration treatments, and the non-tumorigenic *Lars2* and *Dynlt1a* (identified through this approach) are viable candidates for the development of axon regeneration therapies. The experimental approach in another study injected retrograde tracer proximally to the injury site (1.5 mm away), which enabled the capture of many RGCs that regenerate short-distance axons (Jacobi et al., 2022), and did not use comparative analysis with embryonic RGCs as we did in our approach. Therefore, that study revealed different factors and insights than reported here. For example, it showed that primarily (82%) αRGCs (and 18% of multiple other RGC subtypes of which non-α ipRGCs represented only ∼1.8%; see figure 2G in that study) respond to *Pten* KO by regenerating axons short distance, whereas our study revealed that primarily non-α M1 ipRGC subtypes C33 and C40 respond to *Pten* KD by regenerating long-distance axons. Comparative analysis between short-distance axon-regenerating RGCs from that study (Jacobi et al., 2022) and long-distance axon-regenerating RGCs identified herein is shown in Fig. S1. However, this analysis did not include cells from another similar study (Li et al., 2022) that also found short-distance axon-regenerating genes, because retrospective analysis found those cells to be microglia/macrophages (Theune and Trakhtenberg, 2023 preprint). Also, *Gal*, which has been previously implicated in peripheral nervous system axon regeneration (Holmes et al., 2000), was one of the few identified RGC axon regeneration-promoting genes in that study (Jacobi et al., 2022), whereas we identified *Gal* as one of the markers of adult RGC subtypes C33/C40 that retained features of an embryonic cell state, which may have enabled enhanced long-distance axon regeneration in response to *Pten* KD. *Gal* is enriched in RGC subtypes C33/C40 nearly 3-fold relative to αRGCs and even more relative to other RGC subtypes in the uninjured RGC atlas. However, after ONC and *Pten* KO, *Gal* is also upregulated in αRGCs, although these cells did not dedifferentiate towards embryonic cell state.

Our study suggests that the degree of similarity of a neuron to the embryonic transcriptomic state is a predictor of its responsiveness to axon regeneration treatments, and that the adult neuronal subtypes that retain embryonic cell state features may have enhanced long-distance axon regeneration response to treatments. Considering that *Pten* KD downstream factors, Dynlt1a and more so Lars2, achieved axon regeneration ∼full-length of the optic nerve by 2 weeks after injury (despite fewer short-distance regenerating axons compared with *Pten* KD; Yungher et al., 2015; Kim et al., 2018), and considering that targeting other factors in the Pten pathway also promotes axon regeneration (even without complementary co-treatments) (Li et al., 2016; Bray et al., 2019; Lukomska et al., 2021), it is possible that several effectors may promote axon regeneration through tapping into subtype-specific and non-subtype-specific pathways, which could tilt the transcriptome towards an embryonic transcriptomic state. For example, we found that expression of the axon regeneration-facilitating Tet1 demethylase (which is upregulated by a dedifferentiation treatment co-expressing Oct4/Sox2/Klf4/Myc) (Lu et al., 2020) is substantially downregulated in the injured RGCs, but its expression is preserved (and even modestly upregulated) in the long-distance axon-regenerating RGCs that responded to *Pten* KD. Moreover, the Sox2 component of that treatment, which is not expressed in injured or uninjured RGCs, is upregulated in the long-distance axon-regenerating RGCs that responded to *Pten* KD. Furthermore, several other known axon regeneration-promoting genes were also co-upregulated in the long-distance axon-regenerating RGCs by *Pten* KD, including *Braf* (O'Donovan et al., 2014), *Akt3* (Campion et al., 2022), *Igf1R* (Dupraz et al., 2013) and *Sprr1a* (Bonilla et al., 2002).

Taken together, our findings reveal the existence of adult neuronal subtypes that retained features of the embryonic cell state, demonstrate that the RGCs which regenerate long-distance axons in response to *Pten* KD originate from these embryonic-like adult subtypes and dedifferentiate towards an embryonic cell state, and identify novel axon regeneration-promoting genes, which suggest that mitochondrial protein synthesis may be rate-limiting in axon regeneration.

## MATERIALS AND METHODS

### Animal use, surgeries, cell labeling and isolation

All animal studies were performed at the University of Connecticut Health Center with approval of the Institutional Animal Care and Use Committee and the Institutional Biosafety Committee, and performed in accordance with the ARVO Statement for the Use of Animals in Ophthalmic and Visual Research. Mice were housed in the animal facility with a 12 h light/12 h dark cycle (lights on from 07:00-19:00) and a maximum of five adult mice per cage. The study used wild-type 129S1/SvImJ mice (The Jackson Laboratory). Optic nerve surgeries and injections, and intravitreal injections, were carried out on mice of both sexes at 8-12 weeks of age (average body weight 20-26 g) under general anesthesia, as described previously (de Lima et al., 2012; Trakhtenberg et al., 2018; Kim et al., 2018; Lukomska et al., 2021). The viruses included AAV2 vectors expressing anti-Pten shRNA or scrambled shRNA control (both co-expressing mCherry reporter and using published sequences; Yungher et al., 2015; Kim et al., 2018; Lukomska et al., 2021), as well as Dynlt1a [open reading frame (ORF) of ENSMUST00000169415.2], Lars2 (ORF of ENSMUST00000038863.8), and mCherry (titers ∼1×10^12^ GC/mL; VectorBuilder). Viruses (2 μl per eye) were injected intravitreally in 8-week-old mice, avoiding injury to the lens, 2 weeks prior to ONC surgery. Mice were randomly assigned to experimental or control conditions. This lead time allowed for sufficient transduction and expression of the shRNAs and transgenes in RGCs at the time of ONC. Transduction efficiency was ∼30%, which is similar to previous reports that also used AAV2 to target the RGCs (Yungher et al., 2015; Kim et al., 2018; Lukomska et al., 2021; Trakhtenberg et al., 2018, 2014).

To label the RGCs that regenerated axons in response to treatment with anti-Pten shRNA, axonal tracer CTB conjugated to Alexa Fluor 488 dye (C34775, Thermo Fisher Scientific) was injected (1% CTB in 1 μl PBS) into the end of optic nerve, ∼3 mm from the ONC injury site, 12 h before the enrichment of the RGCs from retinal single-cell suspension by immunopanning for Thy1 (as described previously; Trakhtenberg et al., 2014; Rheaume et al., 2018) for FACS (using BD Biosciences FACSAria cell sorter with FACSDiva v8.0 software) at 2 weeks following ONC. Enrichment of RGCs by immunopanning was necessary, because FACS of millions of retinal cells (from 10 retinas) would adversely affect primary neurons such as RGCs, which comprise <1% of all the retinal cell types (Jeon et al., 1998; Masland, 2012; Smith and Chauhan, 2015) (e.g. recovery of RGCs from whole retinas for scRNA-seq was limited to only 432 RGCs from multiple retinas; Macosko et al., 2015). Cells enriched for RGCs by immunopanning were then subject to FACS for mCherry^+^/CTB^+^ cells, and immediately processed by plate-based droplet scRNA-seq (see below). To inject CTB into the end of the optic nerve, the anesthetized mouse skull was exposed, drilled on the Bregma, and cut along the coronal suture line. Then, ∼5 mm width of the bone was removed laterally up to the cortical area, under which the optic nerve enters the optic chiasm. Parts of frontal cortex, striatum, thalamic nuclei and nucleus of the stria terminalis of amygdala were removed with a surgical spatula to expose the optic nerves along the anterior cranial fossa (taking care to minimize damage to the blood vessels) just enough to enable visualization of the optic nerve segment that enters the optic chiasm. To inject CTB, using stereotaxic apparatus, a 50 μm diameter glass needle (pulled from borosilicate glass capillaries BF150-86-10, World Precision Instruments, using Flaming/Brown P-97 micropipette puller) was guided and inserted into the optic nerve segment adjacent to the optic chiasm. After visually confirmed injection, coagulant was added to stop bleeding and the skin was sutured. Standard stereotaxic surgery analgesic regimen was administered. Mice were kept warm using a heating-pad, food and water placed in a Petri dish, and mice were checked regularly through to sacrifice 12 h later.

### Tissue processing

Standard histological procedures were used, as described previously (Kim et al., 2018; de Lima et al., 2012; Kurimoto et al., 2010; Trakhtenberg et al., 2018; Lukomska et al., 2021). Briefly, anesthetized mice were transcardially perfused with isotonic saline followed by 4% paraformaldehyde (PFA) at 2 weeks after ONC, the eyes and optic nerves were dissected, postfixed for 2 h, the retinas were dissected-out and optic nerves were transferred to 30% sucrose overnight at 4°C. The optic nerves were then embedded in OCT Tissue Tek Medium (Sakura Finetek), frozen, cryostat-sectioned longitudinally at 14 µm and then mounted for imaging on coated glass slides. Free-floating retinas were immunostained in 24-well plate wells and, after making four symmetrical slits, flat-mounted on coated glass slides for imaging. For immunostaining, free-floating retinas were blocked with the appropriate sera, incubated overnight at 4°C with primary anti-βIII-Tubulin (1:500, rabbit polyclonal; Abcam, Ab18207) antibody, then washed three times in PBS, incubated with fluorescent secondary antibody (1:500; Alexa Fluor, A21207, Thermo Fisher Scientific) for 4 h at room temperature, washed three times again in PBS and mounted for imaging. Images of the regenerating axons in the optic nerve and surviving RGCs in Fig. 4 were acquired using a fluorescent microscope (Zeiss, AxioObserver.Z1). Retinal flatmount images of RGCs positive for CTB and/or mCherry in Fig. 1 were acquired using a confocal microscope (Zeiss, Confocal LSM800).

### Quantification of regenerated axons and RGC survival

To visualize the regenerating axons or their absence after treating with the viral vectors expressing *Dynlt1a*, *Lars2* or mCherry control, axonal tracer [Alexa Fluor 488-conjugated CTB (C34775, Thermo Fisher Scientific) 1% in 3 μl PBS] was intravitreally injected 1 day before animals were euthanized 2 weeks following ONC. Longitudinal sections of the optic nerve were examined for possible axon sparing (Kim et al., 2018). No spared axons were found in control, and no evidence of axon sparing was found in experimental conditions (i.e. at 2 weeks after injury, no axons were found distal from the injury region of the optic nerve). Regenerated axons (defined as continuous fibers, which are absent in controls and are discernible from background puncta and artefactual structures) were counted manually using a fluorescent microscope (Zeiss, AxioObserver.Z1) in at least four longitudinal sections per optic nerve at 0.5 mm, 1 mm, 1.5 mm, 2 mm and 3 mm distances from the injury site (identified by the abrupt disruption of the densely packed axons near the optic nerve head, as marked by a rhombus in Fig. 4B), and these values were used to estimate the total number of regenerating axons per nerve, as previously described (de Lima et al., 2012; Kim et al., 2018; Trakhtenberg et al., 2018; Lukomska et al., 2021). RGC survival was quantified in retinal flatmounts as previously described (Kim et al., 2018; de Lima et al., 2012; Kurimoto et al., 2010; Trakhtenberg et al., 2018; Lukomska et al., 2021) by immunostaining with an antibody to βIII-Tubulin (neuronal marker Tuj1; encoded by *Tubb3*) and counterstained with DAPI to label the nuclei, taking advantage of the selective expression of βIII-tubulin in RGCs. ImageJ software was used to count βIII-Tubulin^+^ cells from images taken at 1-2 mm from the optic nerve head in four directions, then averaged to estimate overall RGC survival per mm^2^ of the retina. Investigators performing the surgeries and quantifications were masked to the group identity by another researcher until the end of the experiment.

### Plate-based droplet scRNA-seq of *Pten* KD long-distance axon-regenerating RGCs

Custom designed Drop-seq barcodes from Integrated DNA Technologies (IDT) were delivered into wells of two 384-well plates. All primers in one well shared the same unique cell barcode and billions of different unique molecular identifiers (UMIs). An Echo 525 liquid handler was used to sequentially dispense lysis buffer, primers (custom designed Drop-seq barcodes from IDT) and reaction reagents, totaling 1 μl, into each well in the plate for the cell lysis and cDNA synthesis using a modified Drop-seq/SmartSeq2 protocol. Following cDNA synthesis, the contents of each well were collected and pooled into one tube using a Caliper SciClone Liquid Handler. After treatment with exonuclease to remove unextended primers, the cDNA was PCR amplified for 13 cycles and then fragmented and amplified for sequencing using a Nextera XT DNA sample prep kit (Illumina) using custom primers (Table S2) that enabled the specific amplification of only the 3′ ends. Paired-end FASTQs were generated using BCL2FASTQ v2.18.0.12 (Illumina)*.* A digital expression matrix was constructed for each pair of FASTQs using Drop-seq tools v1.13 (http://mccarrolllab.com/dropseq) as follows: Bam creation with Picard (v2.9.3) FastqToSam; cell and UMI tagging, filtering, trimming with Drop-seq tools TagBamWithReadSequenceExtended, FilterBAM, TrimStartingSequence, PolyATrimmer; alignment with STAR (v2.5.4a) to the mm10-1.2.0_genome and transcriptome from CellRanger (for comparisons with 10x Genomics datasets); sorting with Picard (v2.9.3) SortSam; merging and tagging with Picard (v2.9.3) MergeBamAlignment and Drop-seq_tools (v1.13) TagReadWithGeneExon; and a gene-cell expression matrix of raw 3′ end counts (in CSV format) was produced with Drop-seq_tools (v1.13) DigitalExpression. Cells not expressing RGC genes such as *Rbpms* and *Tubb3*, as well as poor quality cells or doublets, were excluded.

### Procurement and initialization of previously generated RGC scRNA-seq datasets

BAM files, raw counts, normalized matrices and cell metadata (e.g. type assignment) were obtained for the mouse embryonic RGCs from Gene Expression Omnibus (GEO) deposit GSE122466 (Lo Giudice et al., 2019), and for the mouse adult RGC atlas and the injured RGCs from the GEO deposit GSE137400 (Tran et al., 2019). BAM files were converted to FASTQ files using CellRanger bamtofastq software. FASTQ files were aggregated where appropriate using CellRanger and then mapped to the CellRanger mm10-1.2.0 transcriptome. Batch correction was performed for separate batches using the FindIntegrationAnchors and IntegrateData functions from Seurat v.4.0.3 (Stuart et al., 2019; Hao et al., 2021). The same cells that passed the original quality checks and the same cell-to-type assignments from the original analyses (Lo Giudice et al., 2019; Tran et al., 2019) were used in the present study. Normalized count matrices were obtained for the mouse *Pten* KO RGCs (120 cells in total) from the GEO deposit GSE202155 (Jacobi et al., 2022). Comparative analysis of scRNA-seq datasets was performed using the R package Seurat v.4.3.0 (see below).

### Bioinformatic analyses of long- and short-distance axon-regenerating RGCs

The plate-based-generated SmartSeq2 scRNA-seq dataset (*Pten* KD long-distance axon-regenerating RGCs) was merged with the 10x Genomics single-cell platform-generated scRNA-seq datasets (embryonic, adult atlas and adult injured RGCs) using SAVER (Huang et al., 2018), with default parameters for the plate-based-generated scRNA-seq, for the downstream comparative analyses between the datasets. All datasets were normalized using Seurat's NormalizeData function with default parameters (Stuart et al., 2019; Hao et al., 2021). The sex-specific genes *Xist*, *Eif2s3y* and *Ddx3y* were excluded for dimensionality reduction but retained for downstream analyses. Embryonic and adult RGCs were aligned using the mutual nearest neighbors algorithm from Batchelor (Haghverdi et al., 2018) as part of the Monocle v.3 (Cao et al., 2019), which was used to generate the merged embryonic and adult atlas RGC UMAP. Monocle was also used to determine the pseudo-timeline structure of the merged (embryonic/adult RGC) UMAP. The UMAP cell embeddings, generated by Monocle, were transferred to a Seurat object containing the same datasets, and the hyperparameters (umap.n.neighbors=10; umap.metric=‘euclidean’; umap.min.dist=0.1; n_epochs=200; learning_rate=1; repulsion_strength=1; negative_sample_rate=5; approx._pow=0; spread=1) were used to generate a Seurat UMAP model. The CellTools algorithm (Rheaume and Trakhtenberg, 2022 preprint) was used to map the *Pten* KD regenerating RGCs to their cell type origins, and the injured and *Pten* KD long-distance axon-regenerating RGCs were individually assigned the same pseudo-timeline score as their nearest reference neighbor. The counts-matrix for the plate-based-generated SmartSeq2 scRNA-seq dataset of the 120 short-distance axon-regenerating RGCs was obtained from the GEO deposit GSE202155 (Jacobi et al., 2022) and merged as above with the 10x Genomics single-cell platform-generated scRNA-seq datasets using SAVER (Huang et al., 2018), with default parameters for the plate-based-generated scRNA-seq for the downstream comparative analyses between the datasets. Normalization, mapping to the reference UMAP and assignment of the pseudo-timeline scores for the short-distance axon-regenerating RGCs was also performed as above. Comparative analysis of scRNA-seq datasets was performed using the R package Seurat v.4.3.0.

### Heatmaps and violin plots

Heatmaps were generated using Superheat (Barter and Yu, 2018), with the average expression of each gene per group scaled using *z*-scores, as previously published (Rheaume et al., 2018). The genes were ordered by the log2 fold-change between average expression in the *Pten* KD long-distance axon-regenerating RGCs and average expression in the injured control RGCs from clusters C33 and C40. Violin plots were generated using ggplot2 and Seurat's VlnPlot function (Stuart et al., 2019; Hao et al., 2021). Expression data for specific genes was extracted using Seurat's VlnPlot function. Violin plots were generated using the ggplot2 geom_violin function, and overlayed categorical scatter (violin point) plots were generated using ggbeeswarm (https://cran.r-project.org/web/packages/ggbeeswarm/).

### Gene-concept network plot and functional enrichment analysis

Functional enrichment analysis was performed using the R package gprofiler2 on genes upregulated (determined using Seurat's FindMarkers function) in *Pten* KD long-distance axon-regenerating RGCs relative to injured untreated RGCs (excluding C33 and C40), with all genes expressed in injured RGCs set as background (Kolberg et al., 2020). False discovery rate (FDR) was used for multiple testing correction. A subset of GO:BP terms containing the genes *Dynlt1a* and *Lars2* were plotted in a Gene-Concept Network Plot using the clusterProfiler and enrichplot R packages (Wu et al., 2021).

### Statistical analyses

All tissue processing, quantification and data analysis were carried out masked throughout the study. Sample sizes were based on accepted standards in the literature and our previous experiences. Sample size (*n*) represents total number of biological replicates in each condition. All experiments included appropriate controls. No cases were excluded in our data analysis, although a few animals that developed a cataract in the injured eye were excluded from the study and their tissues were not processed. The data are presented as mean±s.e.m. and was analyzed (as specified in the applicable figure legends) by ANOVA with or without Repeated Measures and a posthoc LSD test (SPSS). Significance of enrichment fold-change in Fig. 2I was determined using the EdgeR algorithm (McCarthy et al., 2012). Significance for DEGs in the heatmaps in Fig. 3 was determined by independent samples Mann–Whitney *U-*test, using R software as previously published (Rheaume et al., 2018). All differences were considered significant at *P<*0.05.

## Supplementary Material

10.1242/develop.201644_sup1 Supplementary information
