# Supplementary material for: Pten inhibition dedifferentiates long-distance axon-regenerating intrinsically photosensitive retinal ganglion cells and upregulates mitochondria-associated Dynlt1a and Lars2
Source: Development. 2023 Apr 24;150(8):dev201644. doi: 10.1242/dev.201644 (PMC10163351; doi:10.1242/dev.201644)
Supplement: Supplementary information [file develop-150-201644-s1.pdf]

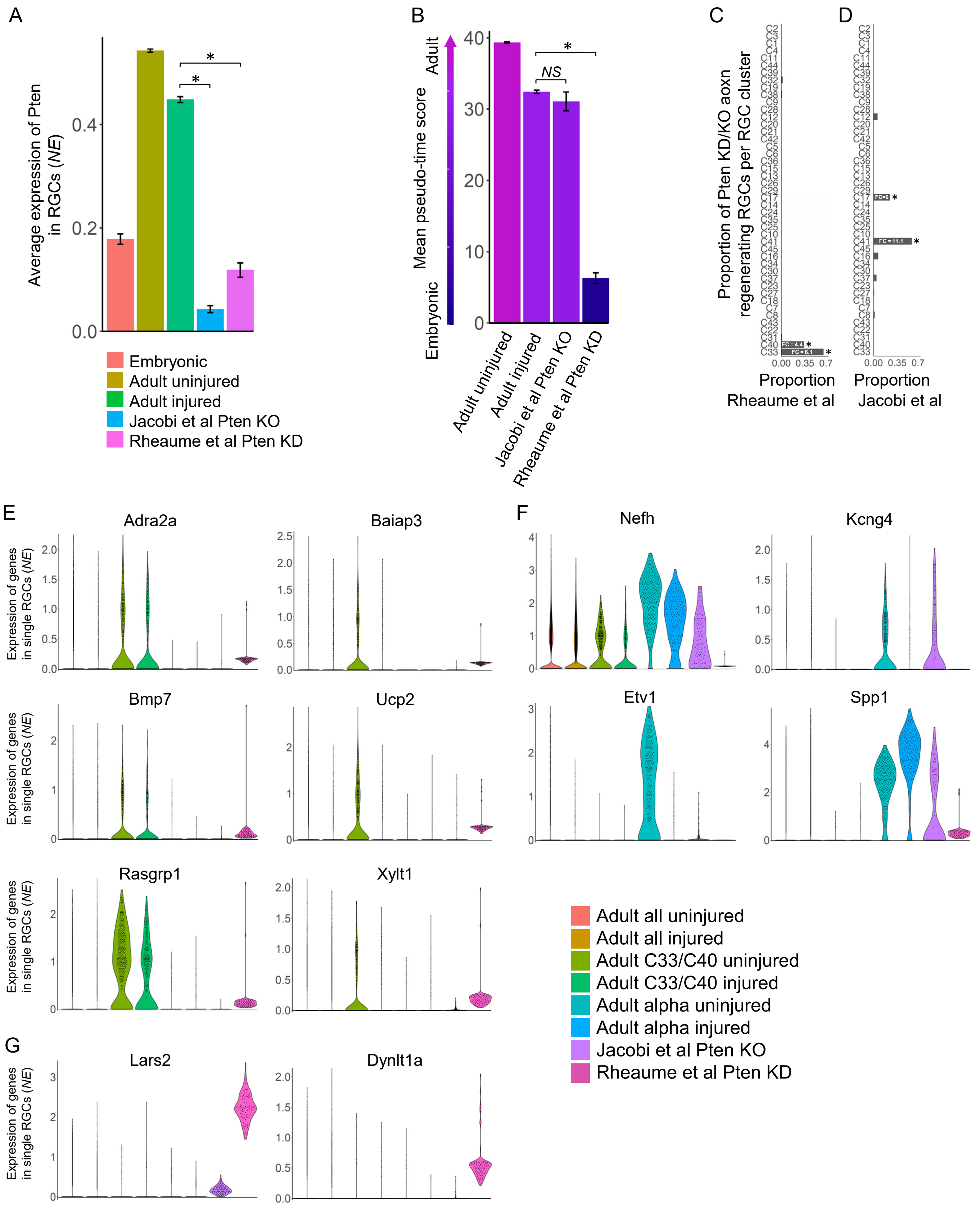

Fig. S1. Pten KO short-distance axon-regenerating RGCs belong to different subtypes than long-distance axon-regenerating RGCs

(**A**) Average gene expression of Pten in RGCs from different conditions, as marked, shows that Pten expression was significantly (* p < 0.001) reduced several folds, compared to untreated RGCs, and also below embryonic level of expression, in the Pten KO RGCs from Jacobi et al. and in the Pten KD RGCs we analyzed (Rheaume et al). Mean ± SEM shown; overall F = 199.5, p < 0.001 by ANOVA, with p-values from pairwise comparisons by posthoc LSD.

Development • Supplementary information

(**B**) Average pseudo-time scores for RGCs from different conditions, as marked, show that pseudo-time score of the Pten KO primarily αRGCs from Jacobi et al is not significantly (p = 0.39) reduced from that of injured untreated RGCs, whereas pseudo-time score of the Pten KD C33/C40 RGCs we analyzed (Rheaume et al) is significantly reduced (* $p < 0.001$), reverting to within embryonic RGC cell state range (consistent with the main Fig. 2G). Mean ± SEM shown; overall $F = 492.1$, $p < 0.001$ by ANOVA, with p-values from pairwise comparisons by posthoc LSD. *NS* = Not Significant.

(**C-D**) Compared to cluster proportion in the adult RGC atlas, the proportion of Pten KD long-distance axon-regenerating RGCs analyzed herein (Rheaume et al), which bioinformatically mapped to adult atlas RGC clusters, is significantly enriched in the adult RGC clusters C33/C40 that are the closest to the embryonic RGC state (on the embryonic-adult RGC state pseudo-timeline, x-axis in main Fig. 2H; $p < 0.05$ by EdgeR indicated by asterisk *, see main Fig. 2I) (*C*). By contrast, the proportion of Pten KO primarily short-distance axon-regenerating RGCs from Jacobi et al, which bioinformatically mapped to adult atlas RGC clusters, is most significantly enriched in the adult αRGC cluster C41, with almost no cells mapping to ipRGCs near the embryonic-like RGC clusters (* $p < 0.05$ by EdgeR) (*D*).

(**E**) M1 ipRGC C33/C40 cluster markers Adra2a, Baip3, Bmp7, Ucp2, Rasgrp1 and Xylt1 (as marked) are enriched in Pten KD long-distance axon-regenerating RGCs analyzed herein (Rheaume et al), but not in the Pten KO (primarily short-distance axon-regenerating) RGCs from Jacobi et al.

(**F**) αRGC markers Nefh, Kcng4, Etv1, and Spp1 (as marked) are enriched in Pten KO RGCs from Jacobi et al (a majority of which mapped to αRGC subtype; *D*). By contrast, none of these αRGC markers are enriched in Pten KD long-distance axon-regenerating RGCs analyzed herein (Rheaume et al), with the exception of low levels of Spp1 expression, which has been reported to be upregulated after Pten KO (see references in the main text).

(**G**) Lars2 and Dynlt1a genes, discovered herein to be downstream of Pten KD in RGCs that regenerated long-distance axons, and sufficient to promote axon regeneration on their own, are upregulated only slightly (Lars2) or not at all (Dynlt1a) in Pten KO RGCs from Jacobi et al (a majority of which are primarily short-distance axon-regenerating αRGCs), but both are highly upregulated in Pten KD long-distance axon-regenerating RGCs (which are ipRGC C33/C40 as reported herein, by Rheaume et al).

Table S1. Genes differentially enriched or unenriched in the Pten KD long-distance axon-regenerating RGCs

Normalized expression (*NE*) levels of the DEGs, which are differentially enriched or unenriched in the Pten KD long-distance axon-regenerating regenerating RGCs compared to their clusters of origin (C33 and C40) in the ONC RGC dataset (upregulated: log2 FC ≥ 1.5 and Pten KD RGC expression ≥ 0.5 *NE*; downregulated: log2 FC ≤ -1.5 and ONC RGC expression ≥ 0.5 *NE*; $p < 0.05$; Mann–Whitney *U* test). Expression of these genes is also shown for embryonic and adult RGCs, as marked.

Click here to download Table S1

Table S2. Custom primers

Oligo dT for RT (ex. Well A1)	[25nt universal anchor (*or PCR handle*) --12nt barcode--8nt UMI--30nt poly-dT]<br>AAGCAGTGGTATCAACGCAGAGTACaacaagtcgctgNNNNNNNNTTTTTTTTTTTTTTTTTTTTTTTTTTTTTTVN
TSO_RT	5'-AAGCAGTGGTATCAACGCAGAGTGAATrGrG+G-3'
TSO_ISPCR_cDNA	5'-AAGCAGTGGTATCAACGCAGAGT-3'
P5-TSO-Hybrid_LibP	5'-AATGATACGGCGACCACCGAGATCTACACGCCTGTCCGCGGAAGCAGTGGTATCAACGCAGAGT*A*C-3'
Custom Read 1 Primer	5'-CGGAAGCAGTGGTATCAACGCAGAGTAC-3'

Development • Supplementary information
