# Supplementary material for: Pten inhibition dedifferentiates long-distance axon-regenerating intrinsically photosensitive retinal ganglion cells and upregulates mitochondria-associated Dynlt1a and Lars2
Source: Development. 2023 Apr 24;150(8):dev201644. doi: 10.1242/dev.201644 (PMC10163351; doi:10.1242/dev.201644)
Supplement: Supplementary file 1 [file develop-150-201644_review_history.pdf]

Development

Pten inhibition dedifferentiates long-distance axon-regenerating intrinsically photosensitive retinal ganglion cells and upregulates mitochondria-associated Dynlt1a and Lars2

Bruce A. Rheaume, Jian Xing, Agnieszka Lukomska, William C. Theune, Ashiti Damania, Greg Sjogren and Ephraim F. Trakhtenberg
DOI: 10.1242/dev.201644

Editor: Steve Wilson

Review timeline
Original submission: 26 January 2023
Editorial decision: 10 March 2023
First revision received: 14 March 2023
Accepted: 22 March 2023

Original submission

Note - this manuscript was transferred to us with referee reports from another journal.

First decision letter

MS ID#: DEVELOP/2023/201644

MS TITLE: PTEN inhibition promotes long-distance axon regeneration by dedifferentiating neurons towards an embryonic state and upregulating mitochondria-associated DYNLT1A and LARS2

AUTHORS: Bruce A Rheaume, Jian Xing, Agnieszka Lukomska, William C Theune, Ashiti Damania, Greg Sjogren, and Ephraim F Trakhtenberg

Apologies that it took longer than I expected to make a decision on this manuscript. I asked one of our editorial Board to look over the manuscript, reviews and your correspondence. Given that they considered the manuscript potentially suitable for the journal and that the study had already undergone revisions, I just asked for one expert review - that I have now received. The referee's comments are appended below, or you can access them online: please go to BenchPress and click on the 'Manuscripts with Decisions' queue in the Author Area.

As you will see, the reviewer makes suggestions for improving the manuscript that should be straightforward to address and so we will be happy to publish your study after you have made the revisions.

Reviewer 1

Advance summary and potential significance to field

Pten deletion appears to be among the strongest individual manipulations that enhance optic nerve regeneration. However, the length of RGC axon regeneration at 2 weeks post optic nerve injury with only Pten inactivation remains low (~1.5mm). Studies indicate that aRGCs are largely responsible for most of the axon regeneration observed with Pten inactivation. Nonetheless, some RGC axons do regenerate longer distances (2-3mm) and it is important to identify these rare RGC subtypes to learn of mechanisms underlying their enhanced regenerative response. Rheaume et al., developed a strategy to purify these rare RGCs. After comparing their transcriptomes

© 2023. Published by The Company of Biologists under the terms of the Creative Commons Attribution License (https://creativecommons.org/licenses/by/4.0/).

(scRNAseq) to previously published RGC subtype transcriptome data, they identify the intrinsically photosensitive (IP) M1 RGC subtype (clusters C33, C40) as responsible for this enhanced regenerative response. These RGC subtypes have gene expression programs that are more similar to embryonic RGCs than adult RGCs, perhaps underlying their enhanced regenerative response, but this was not tested. Further, Pten knockdown induced 2 genes, Dynlt1a and Lars2, in C33, C40 subtypes and overexpression of these genes is shown to stimulate axon regeneration when delivered to RGCs.

Comments for the author

Pten deletion appears to be among the strongest individual manipulations that enhance optic nerve regeneration. However, the length of RGC axon regeneration at 2 weeks post optic nerve injury with only Pten inactivation remains low (~1.5mm). Studies indicate that aRGCs are largely responsible for most of the axon regeneration observed with Pten inactivation. Nonetheless, some RGC axons do regenerate longer distances (2-3mm) and it is important to identify these rare RGC subtypes to learn of mechanisms underlying their enhanced regenerative response. Rheaume et al., developed a strategy to purify these rare RGCs. After comparing their transcriptomes (scRNAseq) to previously published RGC subtype transcriptome data, they identify the intrinsically photosensitive (IP) M1 RGC subtype (clusters C33, C40) as responsible for this enhanced regenerative response. These RGC subtypes have gene expression programs that are more similar to embryonic RGCs than adult RGCs, perhaps underlying their enhanced regenerative response, but this was not tested. Further, Pten knockdown induced 2 genes, Dynlt1a and Lars2, in C33, C40 subtypes and overexpression of these genes is shown to stimulate axon regeneration when delivered to RGCs.
This paper has already been through review at Dev Cell and the authors have addressed most of the concerns brought up during that review. My concerns are more on language than experiments.

Major concerns:
1. I find the term “long-distance axon regeneration” vague and perhaps misleading. The data shows a minority of Dynlt1a and Lars2 overexpressing RGCs to regenerate their axons and for those that do regenerate axons, most only achieve a 1mm extension beyond the injury site, while a much smaller number can extend axons 2-3mm. “Long-distance regeneration” seems to imply that axons are regenerating past the optic chiasm and perhaps into the brain, yet this is not investigated. Perhaps it is more accurate to refer to the consequences of Dynlt1a and Lars2 as enhancing axonal regeneration? The term ‘long-distance” in title, abstract, and throughout the paper seems to exaggerate the effects they are reporting - The results stand on their own without any over-hype.

2. In the Title and Supplementary Discussion of the work the authors state that Pten inhibition promotes long-distance axon…. through partially dedifferentiating towards an embryonic state. I don’t think this paper shows that the observed partial dedifferentiation to an embryonic state or the fact that C33 and C40 clusters are more embryonic like, contribute to the enhanced axonal regeneration, rather they are correlative.

3. The authors claim Dynlt1a and Lars2 promote long-distance axon regeneration.
I don’t see this in the data. All I can discern from the text and figures is that these genes, when overexpressed, stimulate axonal regeneration. They may enhance axon regeneration beyond that observed when Pten is knocked down, yet there is no data in this paper showing a side-by-side comparison with Pten knockdown. Without this data, how can we be sure these genes are enhancing the length of axonal regeneration over that of Pten knockdown. Without this data one could formally argue the method of analysis is why longer regenerating axons are seen and this may be true for Pten knockdown too. This experiment does not have to be done for this paper, but the authors need to temper their conclusions without it.

Minor concerns:
1. Page 2: Please check the first sentence of paragraph 4: “Same-age retinas…… developmental” I cannot understand the sentence; can you state it in another way?
2. Figure 2: why is panel D inserted between panels E-G and panel H? Makes more sense to keep in alphabetical order.
3. Page 4: 2nd paragraph: change “weather” to “whether”.

© 2023. Published by The Company of Biologists under the terms of the Creative Commons Attribution License (https://creativecommons.org/licenses/by/4.0/).

First revision

Author response to reviewers' comments

POINT-BY-POINT RESPONSE TO CRITIQUES

Please find below our response to the Reviewer 1 comments. The reviewer noted issues, which we corrected and thereby improved the manuscript. The revised manuscript addresses all of the issues, as detailed below.

NOTE TO THE REVIEWER: Per the Executive Editor's instruction, in order to make it easier for the readers, we moved most of the supplementary files into the main manuscript, added subheadings in the results section, and changed the manuscript type from a short report to a research article.

REVIEWER 1: Pten deletion appears to be among the strongest individual manipulations that enhance optic nerve regeneration. However, the length of RGC axon regeneration at 2 weeks post optic nerve injury with only Pten inactivation remains low (~1.5mm). Studies indicate that aRGCs are largely responsible for most of the axon regeneration observed with Pten inactivation. Nonetheless, some RGC axons do regenerate longer distances (2-3mm) and it is important to identify these rare RGC subtypes to learn of mechanisms underlying their enhanced regenerative response. Rheaume et al., developed a strategy to purify these rare RGCs. After comparing their transcriptomes (scRNAseq) to previously published RGC subtype transcriptome data, they identify the intrinsically photosensitive (IP) M1 RGC subtype (clusters C33, C40) as responsible for this enhanced regenerative response. These RGC subtypes have gene expression programs that are more similar to embryonic RGCs than adult RGCs, perhaps underlying their enhanced regenerative response, but this was not tested. Further, Pten knockdown induced 2 genes, Dynlt1a and Lars2, in C33, C40 subtypes and overexpression of these genes is shown to stimulate axon regeneration when delivered to RGCs. This paper has already been through review at Dev Cell and the authors have addressed most of the concerns brought up during that review. My concerns are more on language than experiments.

MAJOR CONCERNS:

1. I find the term "long-distance axon regeneration" vague and perhaps misleading. The data shows a minority of Dynlt1a and Lars2 overexpressing RGCs to regenerate their axons and for those that do regenerate axons, most only achieve a 1mm extension beyond the injury site, while a much smaller number can extend axons 2- 3mm. "Long-distance regeneration" seems to imply that axons are regenerating past the optic chiasm and perhaps into the brain, yet this is not investigated. Perhaps it is more accurate to refer to the consequences of Dynlt1a and Lars2 as enhancing axonal regeneration? The term "long-distance" in title, abstract, and throughout the paper seems to exaggerate the effects they are reporting - The results stand on their own without any over- hype.

Response: We edited to tone-down our conclusions. We removed the term "long-distance" from the abstract, and throughout the rest of the manuscript, when describing axon regeneration promoted by Dynlt1a and Lars2. However, when describing the isolation of the rare subset of RGCs which responded to Pten KD by regenerating "long-distance" axons, we retained the term "long-distance" because we isolated specifically the RGCs which regenerate at least the ~full-length of the optic nerve (by injecting retrograde axonal tracer 3 mm distally from the crush site). This is in contrast to other published methodologies, which capture mostly short-distance axon-regenerating RGCs (by injecting retrograde axonal tracer just 1.5 mm distally from the crush site). This distinction of short versus long distance regeneration has important biological consequences (i.e., different RGC subtypes being involved), as we demonstrated in this manuscript. While we did not look in the optic chiasm or in the optic tract past the optic chiasm (which we now acknowledged in the revised discussion and suggested for future long- term studies), the term "long-distance" here refers to the full-length of the optic nerve, and so we have edited to clarify that this is what we meant by saying that we isolated RGCs that regenerated "long-distance" axons (i.e., near full-length of the optic nerve) in response to Pten KD, and therefore also

© 2023. Published by The Company of Biologists under the terms of the Creative Commons Attribution License (https://creativecommons.org/licenses/by/4.0/).

retained it in the title. However, we made sure to remove any mention of “long-distance” claim for axon regeneration achieved by Dynlt1a and Lars2 alone.

2. In the Title and Supplementary Discussion of the work the authors state that Pten inhibition promotes long- distance axon…. through partially dedifferentiating towards an embryonic state. I don’t think this paper shows that the observed partial dedifferentiation to an embryonic state or the fact that C33 and C40 clusters are more embryonic like, contribute to the enhanced axonal regeneration, rather they are correlative.

Response: We edited to tone-down our conclusions. We changed “through” to “associated with” (i.e., correlated) in the title and throughout the manuscript, but we added that it might be “through” and this hypothesis would need to be tested in future studies.

3. The authors claim Dynlt1a and Lars2 promote long-distance axon regeneration. I don’t see this in the data. All I can discern from the text and figures is that these genes, when overexpressed, stimulate axonal regeneration. They may enhance axon regeneration beyond that observed when Pten is knocked down, yet there is no data in this paper showing a side-by-side comparison with Pten knockdown. Without this data, how can we be sure these genes are enhancing the length of axonal regeneration over that of Pten knockdown. Without this data one could formally argue the method of analysis is why longer regenerating axons are seen and this may be true for Pten knockdown too. This experiment does not have to be done for this paper, but the authors need to temper their conclusions without it.

Response: We edited to tone-down our conclusions. When describing axon regeneration promoted by Dynlt1a and Lars2, we removed the term “long-distance” from the abstract, and throughout the rest of the manuscript, along with any implications that it exceeded the axon regeneration achieved by Pten KD. As we did not look in the optic chiasm or in the optic tract past the optic chiasm, we could not reliably arrive at such a conclusion. However, we added a suggestion in the discussion, that future long-term (i.e., longer than the standard 2-week) studies should test this possibility and examine beyond the optic chiasm head-to-head with Pten KD.

MINOR CONCERNS:

1. Page 2: Please check the first sentence of paragraph 4: “Same-age retinas…… developmental” I cannot understand the sentence; can you state it in another way?

Response: We edited to clarify. We meant to say that retinas from same age mice (e.g., E15) contain RGCs at different stages of development, because RGCs are born in waves, and some are born earlier or later than others. That is why it is possible to have a spectrum of transcriptomically maturing RGCs within the same age retina. We explained this just in case someone would wonder why such immature embryonic RGCs segregate into 3 clusters (as we show in Fig. 1), before differentiating into subtypes later after birth. However, it was not the purpose of our paper to characterize the nature of embryonic clusters (e.g., how much of it is explainable by the differences in state of development, or differentiation towards specific subtypes fates). We can remove that sentence altogether if it is distracting.

2. Figure 2: why is panel D inserted between panels E-G and panel H? Makes more sense to keep in alphabetical order.

Response: We wanted to keep alphabetical order, but we would then not be able to keep the arrow from B to its inset shown in F. Technically, we could omit the arrow and just explain in the text of figure legend that the outlined cluster in B is shown in more detail in F inset. However, we thought that the readers would find the arrow helpful. We can do either way, so if removing the arrow in order to maintain alphabetical order is preferred, please let us know and we will rearrange accordingly.

3. Page 4: 2nd paragraph: change “weather” to “whether”.
Response: We have corrected, thank you for noting this typo.

© 2023. Published by The Company of Biologists under the terms of the Creative Commons Attribution License (https://creativecommons.org/licenses/by/4.0/).

Second decision letter

MS ID#: DEVELOP/2023/201644

MS TITLE: Long-distance axon regeneration promoted by PTEN inhibition is associated with dedifferentiating M1 ipRGCs towards an embryonic state and upregulating mitochondria-associated DYNLT1A and LARS2

AUTHORS: Bruce A Rheaume, Jian Xing, Agnieszka Lukomska, William C Theune, Ashiti Damania, Greg Sjogren, and Ephraim F Trakhtenberg
ARTICLE TYPE: Research Article

I am happy to tell you that your manuscript has been accepted for publication in Development, pending our standard ethics checks. Please note that the journal does not allow supplemental discussion and so the office will be in touch with you to resolve this as they edit the manuscript.

© 2023. Published by The Company of Biologists under the terms of the Creative Commons Attribution License (https://creativecommons.org/licenses/by/4.0/).
